# Modulating effect of vitamin D status on serum anti-adenovirus 36 antibody amount in children with obesity: National Food and Nutrition Surveillance

**DOI:** 10.1186/s12887-020-02216-4

**Published:** 2020-06-27

**Authors:** Bahareh Nikooyeh, Bruce W. Hollis, Tirang R. Neyestani

**Affiliations:** 1 Laboratory of Nutrition Research, National Nutrition and Food Technology Research Institute and Faculty of Nutrition Sciences and Food Technology, Shahid Beheshti University of Medical Sciences, Tehran, Iran; 2 Division of Neonatology, Department of Pediatrics, Medical University of South Carolina, Charleston, SC 29425 USA

**Keywords:** Vitamin D, Adenovirus 36, Obesity, Children

## Abstract

**Background:**

The association of ADV-36 infection and obesity has been reported in children.

The objective of this study was to examine the hypothesis that the association between ADV-36 infection and adiposity may be mediated by sub-optimal vitamin D status of the host.

**Methods:**

Ninety one apparently healthy children in different weight categories (normal weight: 33, overweight: 33, obesity: 25) aged 5–18 years were randomly selected from the registered population at National Food and Nutrition Surveillance Program (NFNS). The groups were matched based on age and sex. Anthropometric, biochemical and serological assessments were performed.

**Results:**

The amount of anti-ADV36-Ab increased whereas circulating concentrations of 25(OH) D decreased across BMI categories with higher amounts in children with normal weight than in children with overweight and obesity (31.0 ± 16.4, 22.5 ± 10.5 and 21.9 ± 9.8 nmol/L, respectively, *p* = 0.004). Logistic regression analysis revealed that for each unit increment of anti-ADV36-Ab, the chance of increase in weight was 8.5 times (OR: 8.5, *p* = 0.029). Interestingly, when 25(OH) D was introduced into the model, anti-ADV36-Ab was no longer the predictor of weight increment and the chance of increase in weight reduced 5% for each unit increase in 25(OH) D concentration (OR: 0.95, *p* = 0.012).

**Conclusion:**

It is suggested that ADV36-induced lipogenesis may be mediated by vitamin D deficiency in children with obesity.

## Background

Contributors to childhood obesity are in the “obesogenic environment” of the children including all those aspects in the child’s environment that encourages him or her to eat more (usually unhealthy) foods and to have less physical activity [1]. One of the proposed potential contributors to obesity is suboptimal vitamin D status. Detection of vitamin D receptor (VDR) and its signaling pathways in adipose tissue indicated that vitamin D could potentially affect development, metabolism and functions of body fat mass [2]. Laboratory in vitro studies demonstrated the effect of calcitriol (1,25(OH)_2_D_3_), the active form of the vitamin, and its inactive metabolites on adipogenesis in murine 3 T3-L1 cell line. While VDR knock out (VDR−/−) and 1-α-hydroxylase knock out (CYP27B1 −/−) mice were highly resistant to weight gain due to dietary intake, the over-expression of human VDR in adipose tissue resulted in increased adipogenesis [3]. Interestingly, a cohort study revealed that circulating concentrations of 25(OH) D, the main biomarker of vitamin D status, below 50 nmol/L was related to new onset obesity in adults [4]. Nevertheless, despite some clinical trials showing the fat mass-decreasing and anti-inflammatory effects of supplementary vitamin D in adults [5–7], actually the relationship between vitamin D deficiency and obesity is still like a “chicken and egg story” [8].

Among the causative factors of obesity, a rather newly proposed theory is viral infections [9]. Human adenovirus-36 (ADV-36) through the effect of viral E4orf1 gene on lipogenic enzymes may induce host adipogenesis [10, 11]. The association of ADV-36 infection and obesity has been reported by some research groups in children [12–14] and in adults, as well [15–18]. However, some other studies did not confirm this finding [19, 20] including a study conducted in Iran [21]. One meta-analytical study documented the association between ADV-36 infection and obesity only in adults but not in children [22].

Though the in vitro and in vivo animal studies have reported the association between ADV-36 infection and adipogenesis [23], this association is controversial in humans. In human studies despite statistically significant difference between proportion of seropositive subjects with obesity and seronegative subjects with normal weight, there are usually considerable number of seronegative subjects with obesity and seropositive subjects who are normal weight [24, 25]. Furthermore, the prevalence of overweight/obesity between 1980 and 2013 increased 27.5% for adults and 47.1% for children, with a more remarkable increase in developed countries [26]. This rise can hardly be explained just by ADV-36 infection, which is acquired through intranasal route and respiratory tract [27] and it is expected to be more prevalent in economically poor countries, wherein hunger and underweight are, and probably continue to be, a problem [28].

The other important very noticeable issue is obesity comorbidities. Dysglycemia and diabetes is among the most common obesity complications [29]. It has been estimated that mortality rates due to cardiovascular problems and diabetes would increase by 41 and 21%, respectively, for every 5 unit increase in body mass index above 25 kg/m^2^ [30]. Controversially, ADV-36 infection has been associated with lower dyslipidemia risk [18], increased insulin sensitivity [31] and lower occurrence of diabetes [31]. Even ADV-36 and certain gut microbes have been examined for their ability to ameliorate animal and human blood lipids and glucose [32]. The main question could be “what has protected ADV-36 seropositive normal weight children from adiposity?” One answer could be that seropositve children may be more prone to weight gain in future. At least one prospective study did not confirm this notion [33]. The other answer may be other contributing factors interacting with this association.

We hypothesized that the association between ADV-36 infection and adiposity may be mediated by sub-optimal vitamin D status of the host. To examine this hypothesis, we conducted a case control study on children and adolescents with normal weight, overweight and obesity.

## Methods

Using G*Power 3.1 software (Universität Düsseldorf, Düsseldorf, Germany), the needed total sample size to assure statistical power of 0.8 and effect size of 0.35 was calculated 84 participants. In total, 91apparently healthy children in different weight categories (normal weight: 33, overweight: 33, obesity: 25) aged 5–18 years were randomly selected from the registered population at National Food and Nutrition Surveillance Program (NFNS), a population-based survey conducted periodically in Iran by the National Nutrition and Food Technology Research Institute (NNFTRI) in collaboration with the Deputy of Health of Iran Ministry of Health and Medical Education and United Nations Children’s Fund (UNICEF) as described elsewhere [34]. The groups were matched based on age and sex. The inclusion criteria were having no history of hepatic or renal disease and taking no vitamin D supplements or medications affecting vitamin D metabolism such as anticonvulsant or corticosteroids at least 2 months prior to the study. The study protocol and objectives were clarified for all participants and their parents or their legal guardians before they signed a written informed consent. The study protocol and procedures were approved by Ethics Committee of NNFTRI.

### Anthropometric measurements

Height was measured to the nearest of 0.1 cm using a wall-mounted stadiometer (Seca 206, Seca Company, Hamburg, Germany). Weight was measured while participants wearing light clothing and no shoes with a digital scale accurate to the nearest of 0.1 kg (Seca 840, Seca Company, Hamburg, Germany). Both instruments were calibrated daily. Body mass index (BMI), which has a strong correlation with adiposity in children [35], was defined as weight (kg)/height (m)^2^. Overweight and obesity were categorized using the BMI for age z-score (1–2 and > 2, respectively).

### Laboratory investigations

Blood samples drawn in the morning (08:00–10:00 h) after an overnight fast were centrifuged at room temperature for 10 min at 800 *g* to separate sera, which were then aliquoted and stored at -80 °C immediately until the day of analysis.

Total cholesterol, triglycerides, low-density lipoprotein-cholesterol (LDL-C) and high-density lipoprotein-cholesterol (HDL-C) concentrations were measured by using enzymatic methods (Pars-Azmoon, Tehran, Iran) and an auto-analyzer (Selecta E; Vitalab, Holliston, the Netherlands).

To determine serum concentrations of 25(OH) D, a commercial kit of direct enzyme immune-assay (EIA) was used (DIAsource, Louvain-la-Neuve, Belgium). The intra- and inter-assay variations were 2.5–7.8% (for values of 13.7–203 nmol/L) and 4.3–9.2% (for values 44.25–213.7 nmol/L), respectively, according to the manufacturer’s manual. The accuracy of measurements of 25(OH) D concentrations were ensured using high-performance liquid chromatography [36] in the Laboratory of Nutrition Research, NNFTRI, that has been participating in the Vitamin D External Quality Assessment Scheme (DEQAS) since 2012.

**Fig. 1:**
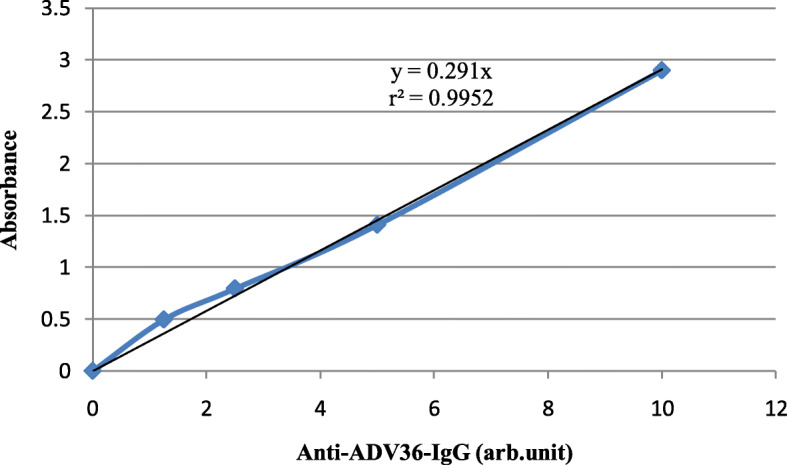
Standard curve of anti-ADV36-Ab

Anti-adenovirus 36 antibody (anti-ADV-36-Ab) was assayed using EIA kit (Zellbio, Veltlinerweg, Ulm, Germany). Briefly, 50 μL pre-diluted serum samples (1:5), control sera (ready to use negative and positive, both included in the kit) followed by enzyme conjugate were transferred to the antibody coated microwells of a plate. After incubation and washing, a color reaction was developed following adding a chromogen to the wells. The reaction was halted using a stop solution. In this assay, the amount of absorbance at 450 nm is proportional to the absolute amount of specific antibody. We generated a standard curve using serially diluted positive control serum. The concentration of anti-ADV-36-Ab was expressed using arbitrary (arb) unit (Fig. 1). The intra- and inter-assay variations were < 10 and < 12%, according to the manufacturer.

### Statistical analyses

Continuous characteristics were expressed as mean ± standard deviation (SD); categorical variables were displayed as frequencies. Normality of distribution was checked for all variables using Shapirio-Wilk test. Tests for differences of continuous variables among three BMI categories were performed using analysis of variance (ANOVA) or Kruskal-Wallis. Levene’s test was used to evaluate homogeneity of between-group variances. For those variables with statistically unequal variances, Dunnett’s T3 was applied for pair wise multiple comparisons. The analysis of covariate test was used to compare anti-ADV-36-Ab among BMI categories by adjusting serum 25(OH) D concentrations. Pearson’s correlation coefficient and multiple linear and logistic regression analysis were used to assess relationships between variables. A 2-tailed *p* < 0.05 was considered significant. Analyses were performed using Statistical Package for Social Sciences 21.0 for Windows (SPSS Inc., Chicago, IL, USA).

## Results

Over 92 and 100% of the participants had undesirable vitamin D status according to Institute of Medicine (IOM) [37] and Endocrine Society Guideline [38], respectively (Table 1). There was no significant difference in anthropometric, biochemical and serum antiviral antibody measures between boys and girls (Table 2). Though circulating 25(OH) D concentrations were higher in boys than in girls, the difference was not statistically significant (27.8 ± 13.0 vs. 22.6 ± 13.3 nmol/L, *p* = 0.068).

**Table 1:** Distribution of vitamin D status in the studied children according to IOM and Endocrine Society criteria

	Deficiency, n (%)	Insufficiency, n (%)	Sufficiency, n (%)
**Institute of Medicine**	52 (57.1)	32 (35.2)	7 (7.7)
**Endocrine Society Practice Guideline**	84 (92.3)	7 (7.7)	0 (0)

Vitamin D status based on circulating 25(OH) D concentrations according to:

a. Institute of Medicine [37]: deficiency < 25 nmol/L; insufficiency: 25–50 nmol/L; sufficiency: > 50 nmol/L

b. Endocrine Society Practice Guideline [38]: deficiency < 50 nmol/L; insufficiency: 50–75 nmol/L; sufficiency: > 75 nmol/L

**Table 2:** Comparison of anthropometric measures, lipid profile components, serum 25(OH) D and anti-ADV36-Ab between boys and girls

Variable	Boys (n_**1**_ = 53)	Girls (n_**2**_ = 38)	***p*** value	Total (***n*** = 91)
**Age (yrs)**	9.16 ± 2.7	10.3 ± 3.5	0.078	9.6 ± 3.1
**Weight (kg)**	42.5 ± 16.9	46.8 ± 22.8	0.308	44.3 ± 19.6
**Height (cm)**	138.4 ± 18.4	138.2 ± 22.5	0.969	138.3 ± 20.1
**BMI (kg/m**^**2**^**)**	21.6 ± 6.1	23.3 ± 6.8	0.215	22.3 ± 6.4
**z-score for BMI for age**	1.33 ± 0.87	1.30 ± 0.94	0.854	1.3 ± 0.9
**Triglyceride (mg/dL)**	87.5 ± 42.4	86.9 ± 47.0	0.954	87.3 ± 44.1
**Total Cholesterol (mg/dL)**	144.3 ± 27.1	148.1 ± 22.7	0.477	145.9 ± 25.3
**HDL-C (mg/dL)**	53.3 ± 13.4	52.0 ± 11.3	0.627	52.7 ± 12.5
**LDL-C (mg/dL)**	73.5 ± 20.8	78.8 ± 18.9	0.220	75.7 ± 21.0
**25(OH) D (nmol/L)**	27.8 ± 13.0	22.6 ± 13.3	0.068	25.7 ± 13.3
**Anti-ADV36-Ab (arb.unit)**	4.05 ± 5.5	3.80 ± 5.5	0.831	3.94 ± 5.5

**Fig. 2:**
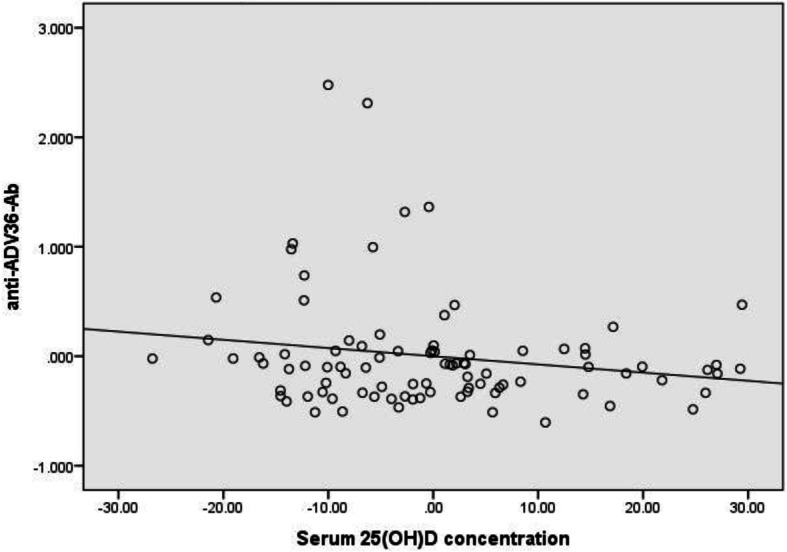
BMI-adjusted association between circulating amounts of 25(OH) D and anti-ADV36-Ab

**Fig. 3:**
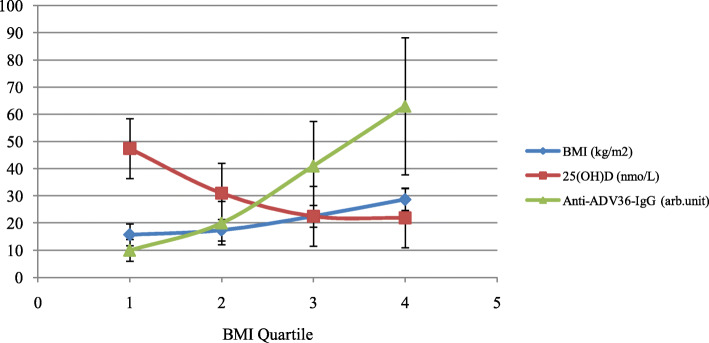
The trends of serum 25(OH) D and anti-ADV-Ab levels across different body mass indices in Iranian 5–18 yr children

Table 3 shows the measures of the studied variables in three BMI categories. The concentrations of anti-ADV36-Ab increased across BMI categories. However, only the difference between categories of normal weight and obesity was statistically significant (2.0 ± 1.6 vs. 6.3 ± 6.8 arb.unit, *p* = 0.013). This difference disappeared in analysis of covariate after adjustment for 25(OH) D concentrations. Circulating concentrations of 25(OH) D decreased across BMI categories with higher amounts in normal weight than in children with overweight and obesity (31.0 ± 16.4, 22.5 ± 10.5 and 21.9 ± 9.8 nmol/L, respectively, *p* = 0.004). Anti-ADV36-Abs showed a direct correlation with BMI z-score (*r* = 0.294, *p* = 0.005) but a negative correlation with circulating 25(OH) D concentrations (*r* = − 0.236, *p* = 0.024) (Fig. 2). Serum 25(OH) D concentrations also inversely correlated with BMI z-score (*r* = − 0.287, *p* = 0.006). Figure 3 shows the trends of serum 25(OH) D and anti-ADV36-Ab levels across BMI quartiles of the studied children.

**Table 3:** Comparison of anthropometric measures, lipid profile components, serum 25(OH) D and anti-ADV36-Ab among children in three weight categories

Variable	Normal Weight (n_**1**_ = 33)	Overweight (n_**2**_ = 33)	Obesity (n_**3**_ = 25)	***p*** value
**Age (yrs)**	9.8 ± 3.1	9.7 ± 3.0	9.3 ± 3.3	0.820
**Weight (kg)**	34.8 ± 13.1^a^	46.0 ± 16.2^b^	53.3 ± 25.3^b^	*0.001*
**Height (cm)**	138.3 ± 19.7	142.0 ± 15.1	133.5 ± 25.7	0.289
**BMI (kg/m**^**2**^**)**	17.4 ± 1.7^a^	22.5 ± 4.1^b^	28.7 ± 7.5^c^	*< 0.001*
**Triglyceride (mg/dL)**	74.9 ± 36.4	90.8 ± 43.0	98.8 ± 52.1	0.104
**Total Cholesterol (mg/dL)**	144.1 ± 24.0	143.3 ± 26.5	151.8 ± 25.5	0.401
**HDL-C (mg/dL)**	55.0 ± 10.2	51.8 ± 13.5	51.0 ± 14.0	0.428
**LDL-C (mg/dL)**	74.1 ± 20.2	73.3 ± 19.3	80.9 ± 20.8	0.308
**25(OH) D (nmol/L)**	31.0 ± 16.4^a^	22.5 ± 10.5^b^	21.9 ± 9.8^b^	*0.004*
**Anti-ADV36-Ab (arb.unit)**	2.0 ± 1.6^a^	4.1 ± 6.4^b^	6.3 ± 6.8^b^	*0.013*

Numbers in a row not sharing a common superscript are significantly different

Logistic regression analysis revealed that for each unit increment of anti-ADV36-Ab, the chance of increase in weight was 8.5 times (OR: 8.5, *p* = 0.029). Interestingly, when 25(OH) D was introduced into the model, anti-ADV36-Ab was no longer the predictor of weight increment and the chance of increase in weight reduced 5% for each unit increase in 25(OH) D concentration (OR: 0.95, *p* = 0.012).

Ordinal regression models were applied to estimate the odds ratios (ORs) and 95% confidence intervals (CIs) of obesity by serum 25(OH) D and anti-ADV36-Ab, adjusted for potential confounders. These models were examined using a full likelihood ratio test comparing the fitted location model to a model with varying location parameters (Table 4). The analysis revealed that with each unit increment of anti-ADV36-Ab, the chance of increase in weight status was 2.86 times (95% CI: 1.25 to 6.52, *p* = 0.012). Interestingly, when 25(OH) D was introduced into the model, anti-ADV36-Ab was no longer the predictor of weight increment and the chance of increase in weight reduced 5% for each unit increase in calcidiol concentration (OR: 0.96, *p* = 0.017).

**Table 4:** Odds Ratio (95% CI) from ordinal regression models for association of weight status with anti-ADV36-Ab

	Variables	OR	95% CI	Wald χ^**2**^	***p*** value
**Model 1**	anti-ADV36-Ab	2.86	1.25 to 6.52	6.24	0.012
**Model 2**	anti-ADV36-Ab	2.27	0.99 to 5.2	3.79	0.052
	25(OH)D	0.961	0.93 to 0.99	5.72	0.017
**Model 3**^**a**^	anti-ADV36-Ab	2.17	0.94 to 5.0	3.35	0.067
	25(OH)D	0.95	0.92 to 0.98	6.64	0.010

^a^ Model 3. Adjusted for sex and age

## Discussion

High occurrence of undesirable vitamin D status in our subjects is in accord with our previous reports [39, 40]. We found an increase in the amount of anti-ADV36-Ab across BMI categories in the studied children indicating a possible effect of subclinical infection in adipogenesis. Several factors have been introduced as the contributors in childhood obesity mostly in the first 1000 days of life and then in the obesogenic environment [41]. Meanwhile, some reports have shown an association between obesity and ADV36 infection [12, 13, 22]. In contrast, some studies failed to show the lipogenic effect of ADV-36 infection [19].

Subclinical viral infections may contribute in metabolic derangements commonly seen in obesity and metabolic syndrome [42]. In a study on Hispanic males and females, the associations among ADV36 seropositivity, adiposity and indicators of glycemic status were examined initially and after almost 10 years. Seropositive subjects showed greater adiposity both at the baseline and after 10 years but they had lower concentrations of fasting serum insulin compared with seronegative subjects. The researchers concluded that ADV36 infection may exert its adipogenic effect even long after the initial infection but may also have a modulatory effect on glycemic control [15]. Improvement of glycemic status in ADV-36 seropositive subjects has been ascribed to the induction of hepatic mitochondrial function [43].

Some meta-analytical studies confirmed the higher risk of adiposity and weight gain in adults due to ADV36 infection [17, 22]. Notwithstanding, we found this association may be modulated by vitamin D status. The inverse relationship of adiposity and circulating 25(OH) D that was observed in this study and other studies [44] may be the key and already missing link between ADV36 infection and obesity. Vitamin D has anti-inflammatory [6], antioxidant [45] and antiviral properties [46]. The exact mechanism of vitamin D antiviral function remains to be clarified but it may pertain to up-regulation of antimicrobial peptides, including cathelcidins (LL-37) and β-defensin 2 [47, 48].

Viruses causing chronic infection with low-grade inflammation including ADV36 may comprise a small part of the host’s metagenome or virome. The final upshot of the relation between the virome and the host could be damaging, harmless or even symbiotic [49] depending on the host’s immunocompetence status. Vitamin D functions as an immunomodulator in several ways by affecting both innate and adaptive immunity [50, 51]. Vitamin D deficiency may, therefore, turn the relation between ADV36 virome and the affected child to damaging by rendering ADV36-induced adipogenesis.

The escalating trends of BMI in children have reached a steady high state in many developed countries but have hastened in certain parts of Asia [52]. These data together with emerging evidence for the relationship between ADV36 infection and obesity have been provocative enough that even vaccination against adenoviral infection has been proposed to induce herd immunity against obesity and its related metabolic derangements [53]. Our findings, however, strengthen the possibility that improvement of vitamin D status of children through supplementation and food fortification might work as well as, and even better than, a true vaccine. Some recent studies support this notion [54–56].

Some limitations in this study are acknowledged. The specificity of EIA method to detect neutralizing anti-ADV-Abs has been questioned [57]. The results of this study need to be confirmed by further studies in other populations with a larger sample size and a more specific method.

## Conclusion

To the best of our knowledge, this the first report of the relation of vitamin D status with anti-ADV36-Ab in children with obesity. We propose that in the future human studies on the association of ADV-36 infection and adiposity, vitamin D status of the subjects must also be taken into consideration. This observational study sets the stage for conducting a randomized controlled trial that would clearly define this proposed relationship.

## Data Availability

The datasets used and/or analyzed during the current study are available from the corresponding author on reasonable request.

## Abbreviations

ADV-36: Adenovirus-36
ANOVA: Analysis of variance
Anti-ADV-36-Ab: Anti-adenovirus 36 antibody
Arb.unit: Arbitrary unit
BMI: Body mass index
CI: Confidence interval
EIA: Enzyme immunoassay
HDL-C: High-density lipoprotein-cholesterol
IOM: Institute of Medicine
LDL-C: Low-density lipoprotein-cholesterol
NFNS: National Food and Nutrition Surveillance Program
NNFTRI: National Nutrition and Food Technology Research Institute
25(OH)D: 25-hydroxycalciferol
OR: Odds ratio
SD: Standard deviation
UNICEF: United Nations Children’s Fund
VDR: Vitamin D receptor

## References

[1] Lake AA (2018). Neighbourhood food environments: food choice, foodscapes and planning for health. Proc Nutr Soc.

[2] Ding C, Gao D, Wilding J, Trayhurn P, Bing C (2012). Vitamin D signalling in adipose tissue. Br J Nutr.

[3] Mutt SJ, Hyppönen E, Saarnio J, Järvelin MR, Herzig KH. Vitamin D and adipose tissue—more than storage. Front Physiol. 2014;5:228. 10.3389/fphys.2014.00228 PMC4067728 25009502

[4] Mai XM, Chen Y, Camargo CA, Langhammer A (2012). Cross-sectional and prospective cohort study of serum 25-hydroxyvitamin D level and obesity in adults: the HUNT study. Am J Epidemiol.

[5] Nikooyeh B, Neyestani TR, Farvid M (2011). Daily consumption of vitamin D- or vitamin D + calcium-fortified yogurt drink improved glycemic control in patients with type 2 diabetes: a randomized clinical trial. Am J Clin Nutr.

[6] Neyestani TR, Nikooyeh B, Alavi-Majd H (2012). Improvement of vitamin D status via daily intake of fortified yogurt drink either with or without extra calcium ameliorates systemic inflammatory biomarkers, including adipokines, in the subjects with type 2 diabetes. J Clin Endocrinol Metab.

[7] Nikooyeh B, Neyestani TR, Zahedirad M (2016). Vitamin D-fortified bread Is as effective as supplement in improving Vitamin D status: a randomized clinical trial. J Clin Endocrinol Metab.

[8] Peterson CA, Belenchia AM (2014). Vitamin D Deficiency & Childhood Obesity: a tale of two epidemics. Mo Med.

[9] Ponterio E, Gnessi L (2015). Adenovirus 36 and obesity: an overview. Viruses.

[10] Nam JH, Na HN, Atkinson RL, Dhurandhar NV (2014). Genomic stability of adipogenic human adenovirus 36. Int J Obes.

[11] Sohrab SS, Kamal MA, Atkinson RL, Alawi MM, Azhar EI (2017). Viral infection and obesity: current status and future prospective. Curr Drug Metab.

[12] Karamese M, Altoparlak U, Turgut A, Aydogdu S, Karamese SA (2015). The relationship between adenovirus-36 seropositivity, obesity and metabolic profile in Turkish children and adults. Epidemiol Infect.

[13] Kocazeybek B, Dinc HO, Ergin S (2017). Evaluation of Adenovirus-36 (Ad-36) antibody seropositivity and adipokine levels in obese children. Microb Pathog.

[14] Park S, Kim J, Shin HJ (2015). Tracking study about adenovirus 36 infection: increase of adiposity. J Microbiol Biotechnol.

[15] Lin WY, Dubuisson O, Rubicz R (2013). Long-term changes in adiposity and glycemic control are associated with past adenovirus infection. Diabetes Care.

[16] Shang Q, Wang H, Song Y (2014). Serological data analyses show that adenovirus 36 infection is associated with obesity: a meta-analysis involving 5739 subjects. Obesity (Silver Spring).

[17] Yamada T, Hara K, Kadowaki T (2012). Association of adenovirus 36 infection with obesity and metabolic markers in humans: a meta-analysis of observational studies. PLoS One.

[18] Almgren M, Atkinson R, He J (2012). Adenovirus-36 is associated with obesity in children and adults in Sweden as determined by rapid ELISA. PLoS One.

[19] Berger PK, Pollock NK, Laing EM (2014). Association of adenovirus 36 infection with adiposity and inflammatory-related markers in children. J Clin Endocrinol Metab.

[20] Broderick M, Hansen C, Irvine M (2010). Adenovirus 36 seropositivity is strongly associated with race and gender, but not obesity, among US military personnel. Int J Obes.

[21] Ehsandar S, Zarkesh M, Daneshpour M, Bandehpour M, Azizi F, Hedayati M (2014). Prevalence of human adenovirus 36 and its association with overweight/obese and lipid profiles in the Tehran lipid and glucose study. Iranian J Endocrinol Metab.

[22] Xu MY, Cao B, Wang DF (2015). Human adenovirus 36 infection increased the risk of obesity: a meta-analysis update. Medicine (Baltimore).

[23] Whigham LD, Israel BA, Atkinson RL (2006). Adipogenic potential of multiple human adenoviruses in vivo and in vitro in animals. Am J Phys Regul Integr Comp Phys.

[24] Atkinson RL (2011). Human adenovirus-36 and childhood obesity. Int J Pediatr Obes.

[25] Aldhoon-Hainerova I, Zamrazilova H, Atkinson RL (2014). Clinical and laboratory characteristics of 1179 Czech adolescents evaluated for antibodies to human adenovirus 36. Int J Obes.

[26] Ng M, Fleming T, Robinson M (2014). Global, regional, and national prevalence of overweight and obesity in children and adults during 1980–2013: a systematic analysis for the global burden of disease study 2013. Lancet.

[27] Hwang KA, Park S, Ahn JH, Nam JH (2018). Development of a standard protocol for quantitative polymerase chain reaction to detect adenovirus 36, which is associated with obesity. Acta Virol.

[28] Risk NC. Factor Collaboration (NCD-RisC). Trends in adult body-mass index in 200 countries from 1975 to 2014: a pooled analysis of 1698 population-based measurement studies with 19.2 million participants. Lancet. 2016;387(10026):1377–96. 10.1016/S0140-6736(16)30054-X PMC7615134 27115820

[29] Guh DP, Zhang W, Bansback N, Amarsi Z, Birmingham CL, Anis AH (2009). The incidence of co-morbidities related to obesity and overweight: a systematic review and meta-analysis. BMC Public Health.

[30] Collaboration PS (2009). Body-mass index and cause-specific mortality in 900 000 adults: collaborative analyses of 57 prospective studies. Lancet.

[31] Almgren M, Atkinson RL, Hilding A (2014). Human adenovirus-36 is uncommon in type 2 diabetes and is associated with increased insulin sensitivity in adults in Sweden. Ann Med.

[32] Dhurandhar NV, Geurts L, Atkinson RL (2013). Harnessing the beneficial properties of adipogenic microbes for improving human health. Obes Rev.

[33] Sabin MA, Burgner D, Atkinson RL (2015). Longitudinal investigation of adenovirus 36 seropositivity and human obesity: the cardiovascular risk in young Finns study. Int J Obes.

[34] Nikooyeh B, Abdollahi Z, Hajifaraji M (2017). Vitamin D status and cardiometabolic risk factors across latitudinal gradient in Iranian adults: national food and nutrition surveillance. Nutr Health.

[35] Katzmarzyk PT, Barreira TV, Broyles ST (2015). Association between body mass index and body fat in 9-11-year-old children from countries spanning a range of human development. Int J Obes Suppl.

[36] Neyestani TR, Gharavi A, Kalayi A (2007). Determination of serum 25-hydroxy cholecalciferol using high-performance liquid chromatography: a reliable tool for assessment of vitamin D status. Int J Vitam Nutr Res.

[37] Rosen CJ, Abrams SA, Aloia JF, Brannon PM, Clinton SK, Durazo-Arvizu RA (2012). IOM committee members respond to Endocrine Society vitamin D guideline. J Clin Endocrinol Metab.

[38] Holick MF, Binkley NC, Bischoff-Ferrari HA, Gordon CM, Hanley DA, Heaney RP (2011). Evaluation, treatment, and prevention of vitamin D deficiency: an Endocrine Society clinical practice guideline. J Clin Endocrinol Metab.

[39] Neyestani TR, Hajifaraji M, Omidvar N, Eshraghian MR, Shariatzadeh N, Kalayi A (2012). High prevalence of vitamin D deficiency in school-age children in Tehran, 2008: a red alert. Public Health Nutr.

[40] Nikooyeh B, Abdollahi Z, Hajifaraji M, Alavi-Majd H, Salehi F, Yarparvar AH (2017). Vitamin D status, latitude and their associations with some health parameters in children: national food and nutrition surveillance. J Trop Pediatr.

[41] Christoffel KK, Wang X, Binns HJ (2012). Early origins of child obesity: bridging disciplines and phases of development -- September 30--October 1, 2010. Int J Environ Res Public Health.

[42] Neyestani T. Immune Alterations in Metabolic Syndrome: The Old Story of Chicken and Egg. In: Bioactive Food as Dietary Interventions for Arthritis and Related Inflammatory Diseases. San Diego: Academic Press; 2013. p. 431–50.

[43] Na HN, Hong YM, Ye MB, Park S, Kim IB, Nam JH (2014). Adenovirus 36 attenuates weight loss from exercise but improves glycemic control by increasing mitochondrial activity in the liver. PLoS One.

[44] Nikooyeh B, Neyestani TR, Alavi-Majd H (2014). Vitamin D deficiency is associated with the metabolic syndrome in subjects with type 2 diabetes. Nutr Food Sci Res.

[45] Nikooyeh B, Neyestani TR (2016). Oxidative stress, type 2 diabetes and vitamin D: past, present and future. Diabetes Metab Res Rev.

[46] Zdrenghea MT, Makrinioti H, Bagacean C, Bush A, Johnston SL, Stanciu LA. Vitamin D modulation of innate immune responses to respiratory viral infections. Rev Med Virol. 2017;27(1):e1909. 10.1002/rmv.1909 27714929

[47] Beard JA, Bearden A, Striker R (2011). Vitamin D and the anti-viral state. J Clin Virol.

[48] Borella E, Nesher G, Israeli E, Shoenfeld Y (2014). Vitamin D: a new anti-infective agent?. Ann N Y Acad Sci.

[49] Virgin HW, Wherry EJ, Ahmed R (2009). Redefining chronic viral infection. Cell.

[50] Skrobot A, Demkow U, Wachowska M (2018). Immunomodulatory role of Vitamin D: a review. Adv Exp Med Biol.

[51] del Giudice MM, Indolfi C, Strisciuglio C (2018). Vitamin D: Immunomodulatory aspects. J Clin Gastroenterol.

[52] NCD Risk Factor Collaboration (NCD-RisC). Worldwide trends in body-mass index, underweight, overweight, and obesity from 1975 to 2016: a pooled analysis of 2416 population-based measurement studies in 128·9 million children, adolescents, and adults. Lancet. 2017;390(10113):2627–42. 10.1016/S0140-6736(17)32129-3. 10.1016/S0140-6736(17)32129-3 PMC5735219 29029897

[53] Na HN, Nam JH (2014). Proof-of-concept for a virus-induced obesity vaccine; vaccination against the obesity agent adenovirus 36. Int J Obes.

[54] Perna S. Is Vitamin D supplementation useful for weight loss programs? A systematic review and meta-analysis of randomized controlled trials. Medicina. 2019;55(7):368. 10.3390/medicina55070368 PMC6681300 31336940

[55] Subih HS, Zueter Z, Obeidat BM, Al-Qudah MA, Janakat S, Hammoh F (2018). A high weekly dose of cholecalciferol and calcium supplement enhances weight loss and improves health biomarkers in obese women. Nutr Res.

[56] Szlagatys-Sidorkiewicz A, Brzezinski M, Jankowska A, Metelska P, Slominska-Fraczek M, Socha P (2017). Long-term effects of vitamin D supplementation in vitamin D deficient obese children participating in an integrated weight-loss programme (a double-blind placebo-controlled study) - rationale for the study design. BMC Pediatr.

[57] Dubuisson O, Day RS, Dhurandhar NV (2015). Accurate identification of neutralizing antibodies to adenovirus Ad36, −a putative contributor of obesity in humans. J Diabetes Complicat.

